# Procyanidins: From Agro-Industrial Waste to Food as Bioactive Molecules

**DOI:** 10.3390/foods10123152

**Published:** 2021-12-20

**Authors:** Leidy Johana Valencia-Hernandez, Jorge E. Wong-Paz, Juan Alberto Ascacio-Valdés, Mónica L. Chávez-González, Juan Carlos Contreras-Esquivel, Cristóbal N. Aguilar

**Affiliations:** 1 Bioprocesses and Bioproducts Research Group, Food Research Department, School of Chemistry, Universidad Autónoma de Coahuila, Unidad Saltillo, Saltillo C.P. 25280, CH, Mexico; johana.valencia@uadec.edu.mx (L.J.V.-H.); alberto_ascaciovaldes@uadec.edu.mx (J.A.A.-V.); monicachavez@uadec.edu.mx (M.L.C.-G.); carlos.contreras@uadec.edu.mx (J.C.C.-E.); 2 Tecnológico Nacional de México, Instituto Tecnológico de Ciudad Valles, Ciudad Valles C.P. 79010, SL, Mexico; jorge.wong@tecvalles.mx

**Keywords:** procyanidins, agro-industrial waste, extraction process, bioactive

## Abstract

Procyanidins are an important group of bioactive molecules known for their benefits to human health. These compounds are promising in the treatment of chronic metabolic diseases such as cancer, diabetes, and cardiovascular disease, as they prevent cell damage related to oxidative stress. It is necessary to study effective extraction methods for the recovery of these components. In this review, advances in the recovery of procyanidins from agro-industrial wastes are presented, which are obtained through ultrasound-assisted extraction, microwave-assisted extraction, supercritical fluid extraction, pressurized fluid extraction and subcritical water extraction. Current trends focus on the extraction of procyanidins from seeds, peels, pomaces, leaves and bark in agro-industrial wastes, which are extracted by ultrasound. Some techniques have been coupled with environmentally friendly techniques. There are few studies focused on the extraction and evaluation of biological activities of procyanidins. The identification and quantification of these compounds are the result of the study of the polyphenolic profile of plant sources. Antioxidant, antibiotic, and anti-inflammatory activity are presented as the biological properties of greatest interest. Agro-industrial wastes can be an economical and easily accessible source for the extraction of procyanidins.

## 1. Introduction

Tannins are a class of heterogeneous phytochemicals of high molecular weight (500–3000 Daltons). They represent an important niche market for the food and chemical industry due to their biological potential [1]. Currently, tannins represent an important alternative in the prevention of chronic and degenerative diseases in humans [2,3]. These compounds are produced as a defense strategy of the plant against biotic or abiotic stress factors [1]. In addition, they are associated with growth and development, and reproduction functions in the plant [2,4,5]. Tannins are categorized into two groups: hydrolysable and condensed tannins, according to their chemical structure withrespect to the presence of aromatic rings, the number of carbon atoms, and the types of bonds [2,3,4].

In recent years, condensed tannins, especially procyanidins, have gained importance. These compounds are abundant in nature and are found in vegetables, fruits, legumes, cereals, and seeds of different plant species [6]. In the literature, they are also known as polyphenols with potent biological activities, including anti-carcinogenic, antimicrobial, antiviral, anti-inflammatory, anti-allergic, antimutagenic, and antihyperglycemic effects; they also help to prevent obesity and heart disease problems [7,8,9,10,11,12].

Agro-industrial wastes are a promising source of procyanidin. Currently, they are discarded at each stage of the food chain. It is estimated that 1.3 billion tons of waste are discarded, mainly pulp, peel, seed, leaf, stem, bark, and others [13]. Disposal is a significant problem in the industry due to environmental pollution and high costs of handling and transport. To overcome these drawbacks, composting, landfilling, feed, and incineration has been explored [14,15]. However, these wastes have bioactive compounds with potential in pharmaceutical, chemical, and food industries, but sustainable processes at low cost [15] are required for extraction.

Degradative and nondegradative methods have been applied to obtain procyanidins [16]. Procyanidins can be in a soluble or insoluble form, and aqueous solvents can extract the first group. Procyanidins in second group, termed nonextractable, are linked to macromolecules of the cell wall as proteins by glycosidic or ester bonds, for which chemical enzymatic and microbiological methods have been investigated [17,18]. Soluble and low molecular weight procyanidins can be extracted through traditional methods such as maceration, thermal reflux, and organic solvents [19,20]. Disadvantages of these processes include high solvent consumption, highly laborious procedures, long extraction times, and risks to human health [21,22].

Nontraditional extraction methods for nonextractable procyanidins include microwave-assisted extraction, ultrasound-assisted extraction, supercritical fluid extraction, and pressurized liquid extraction, among others. These novel extraction methods are also called green, clean or environmentally friendly methods due to their low solvent consumption and short extraction times, and they also present high compound selectivity and higher compound recovery [22,23,24].

Procyanidins are considered to be antinutritional compounds of great diversity and structural complexity, which has led to their limited use in the food, agricultural and livestock sectors [25]. For this reason, new recovery processes for obtaining value-added products from natural economic sources have been advanced. 

The current survey was performed using academic search systems, including Google Scholar, ScienceDirect, PubMed, Scopus and Web of Science. The search was restricted to only articles in English, where a screening by titles and abstracts was carried out according to the topics of our interest. Inclusion criteria were procyanidins, condensed tannins, agro-industrial wastes, biological activity, and extraction methods. Exclusion criteria were articles on only traditional extraction methods or plant material that is not considered agro-industrial wastes.

In studies published within the years 2009 and 2020, using several keywords including “procyanidins”, “agro-industrial wastes”, “extraction methods”, “bioactive compounds”, “structure” and “biological activity” two central axes were found: procyanidin extraction by extraction type or polyphenolic profile by type of agro-industrial waste and its biological potential. The first axis describes various extraction methods, including solid phase extraction, enzyme-assisted extraction, ultrasound-assisted extraction (UAE), microwave-assisted extraction (MAE), accelerated solvent extraction (ASE), pressurized liquid extraction (PLE), hot extraction (HE), and heat reflux (HR). The UAE method has been coupled with the enzymatic and percolation method, and the enzymatic method has been carried out with the help of filtration processes. In addition, comparisons are presented between the conventional methods (infusion, maceration, percolation) and the environmentally friendly methods (UAE, MAE, ASE, HE, HR). In this axis three subcategories are presented: effects of extraction variables, optimization of extraction variables and biological activity of these compounds.

The second axis of major interest is the identification, quantification, and analysis of polyphenolic compounds. Several studies mainly used words such as “identification and quantification of polyphenols”, “investigation of the phytochemical composition”, “characterization of the polyphenolic profile” “determination of the chemical composition” “study of the polyphenolic composition” in their proposed objectives. Five subcategories can be detailed in these investigations: biological activity, cytotoxic effects, nutritional composition, method of extraction, industrial or pharmaceutical application. However, there are few documents that have the study of procyanidins as a central objective [26,27,28,29]. Other studies present the analysis and extraction of catechin, epicatechin, flavan-3-ols, proanthocyanidins, flavonoids, tannins, and condensed tannins, as part of their central objective [29,30,31,32]. From all reviewed papers, only extraction studies of procyanidins and their monomers were included. When the information was poor, studies of condensed tannins were accepted. All the research was on procyanidins or condensed tannins from agro-industrial residues.

Therefore, a review concerning the extraction of procyanidins is required to promote the valorization of agro-industrial wastes. This paper has two main directions. The first is to identify and discuss possible renewable sources of procyanidins and their biological potential. The second is to identify and discussed environmentally friendly extraction strategies for these compounds obtained from agro-industrial wastes. The information found suggests fields for further study and encourages research to continue exploring the discovery of new chemical structures from renewable sources.

## 2. Polyphenols and Procyanidins: Classification and Chemical Structure

Polyphenols comprise one of the most dominant classes of antioxidant bioactive compounds in nature, also known as phenolic compounds [33]. Usually, the phenolic compounds are distinguished on the base of high molecular weight, presence of hydroxyl groups and aromatic rings, number of carbon atoms, and type of bond [1]. They are classified as flavonoids, phenolic acids, lignans, and stilbenes [34]. Among these, the flavonoids are further divided into six subclasses: flavones, isoflavones, flavanols, flavanones, anthocyanins, and proanthocyanidins [35]. The latter group can also be found in oligomeric or polymeric form, and are soluble or insoluble in water, respectively [36]. The proanthocyanidins consists of structures built by catechin, epicatechin and its gallated forms, which are subdivided into three groups, those consisting of (–)-epiafzelequin are called propelargonidins; and those containing (+)-afzelechin are called prodelfinidins, and procyanidins [3,37]. These categories are proposed according to their degree of oxidation and substitution pattern [38].

Other authors report two main groups of polyphenols: hydrolysable and condensed tannins. Hydrolysable tannins, or polyphenolic nonflavonoids, contain hydroxyl groups that are esterified with gallic or ellagic acid. They have a glucose or polysaccharide molecule in their structure [39]. Condensed tannins, also known as proanthocyanidins, are, after lignin, one of the most abundant groups of phenolic compounds in plants [40,41]. They are characterized by having aromatic rings with hydroxyl groups that allow interactions with proteins and carbohydrates through hydrogen bridges. These interactions dependent of bond types (interflavanics), molar mass, galloyl groups and modification of the phenyl hydroxyl groups [40,42]. Because of this, different conformations can be formed in the procyanidin structure, resulting in highly complex molecules with different functional characteristics [3,4,43].

Procyanidins are derived from the group of proanthocyanidins, being the most prominent of this group in plants [44]. They are considered heterogenous compounds due to their diverse chemical structure [45]. These molecules contain flavan-3-ol monomers as basic units in their structure, which are composed exclusively of (+) catechin and (–) epicatechin. (Epi)catechin monomers may be biosynthetic precursors of procyanidins [46,47,48,49]. The type of monomer formed depends on the type of residue present in the structure, such as resorcinol and phloroglucinol, and are present in ring A, with catechol or pyrogallol in-ring B [5]. These compounds can be classified according to chemical structure, hydroxylation pattern and stereochemistry, into oligomers and polymers that can be grouped in three groups by their degree of polymerization (DP), i.e., monomers with DP equal to 1, oligomers with DPs between 2 and 10 and the polymers with DPs higher than 10 [50]. Thus, according to the DP there are monomers, dimers, trimers, and tetramers of procyanidins, corresponding to 1, 2, 3 and 4 base units, respectively [3,42,44]. Among the dimeric and trimeric procyanidins, A1, B1, B2, B3, B4, B8, C1 and T2 have been identified, the last two belonging to the group of trimeric procyanidins [40].

**Figure 1:**
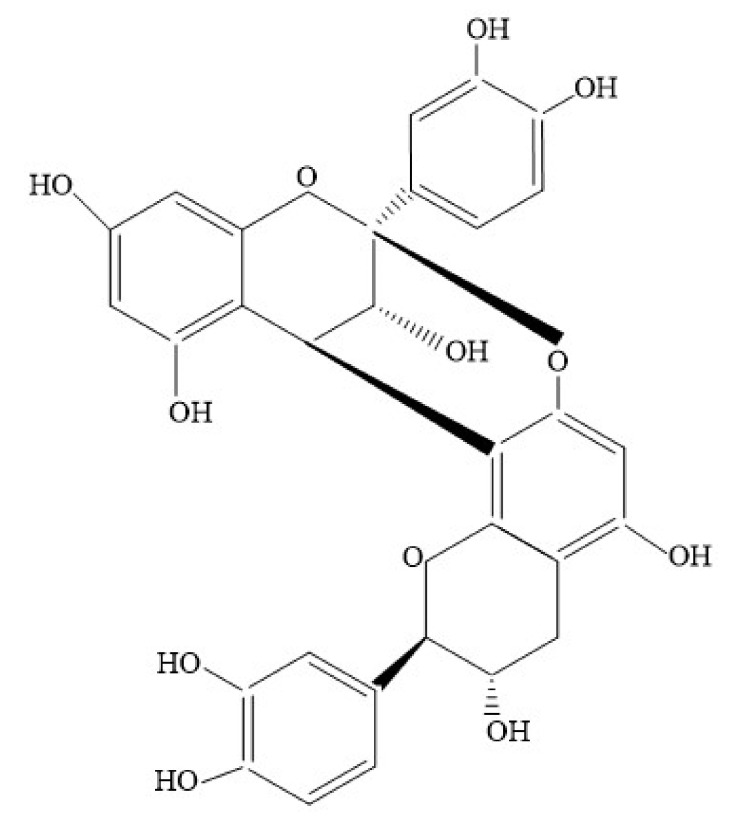
Structure of A1-type dimer procyanidins linked with double C4 → C8 and C2-O-C7 linkage.

**Figure 2:**
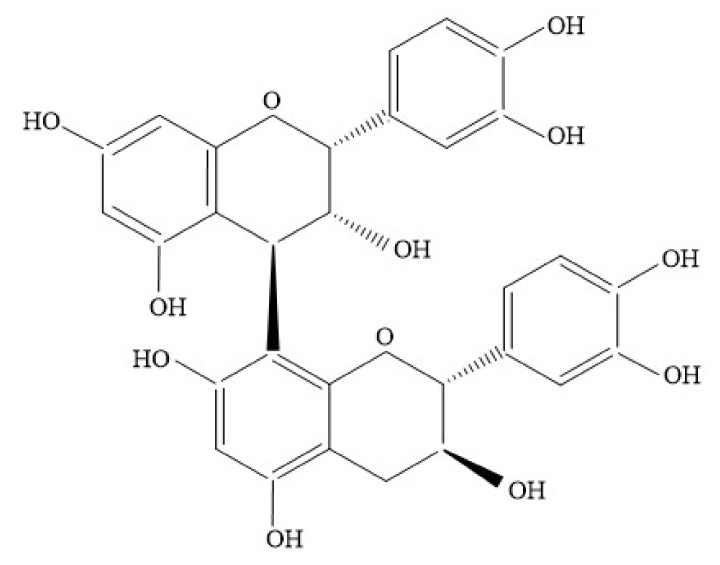
Structure of B1-type dimer procyanidin linked with a single C4 → C8.

Procyanidins possess interflavan bonds in the asymmetric carbons C2 and C3, C4-C8 and C4-C6 [49]. According to the nature of the bond, they can be further classified into type A and B procyanidins. A-type procyanidins contain a double interflavanoid linkage C-C (C4-C8/C6) and an additional ether bond (C2–O–C7 or C2–O–C5); these procyanidins may result from additional oxidation reactions of B-type procyanidin dimers [36,43,51,52]. B-type procyanidins contain interflavonic bonds that are covalently linked in carbon C-4–C-8 or C-4–C-6, where the latter are more prevalent than A-type procyanidins. Figure 1 and Figure 2 shows examples of dimeric procyanidins of type A and B, respectively.

## 3. Agro-Industrial Wastes and Procyanidins

### 3.1. Generation of Agro-Industrial Wastes

The idea of zero-waste has been proposed in production chains, and various investigations have indicated the conversion of these wastes to value-added by-products such as antioxidants, prebiotic ingredients, antimicrobial agents, enzymes, proteins, colorants, bioplastics, and biofuels [45]. The food and nonfood processing industry generates many agro-industrial wastes, including agricultural vegetal and forestry wood, estimated at 55 million tons in 2016 for the European Union [53]. In developed countries, waste is calculated at approximately 0.5 million tons of fruits and vegetables generated in the food industry and establishments serving and distributing. Moreover, fruit and vegetable waste represents 60% of the waste generated annually worldwide [54]. These residues have a wide range of polyphenolic compounds with biological activities [55]. Knowledge of the availability and quantity of these vegetal materials is indispensable for the development and research of reuse strategies. However, accurate data on all waste generated worldwide in each production process remains to be explored [56].

Several studies have been conducted to extract and recover bioactive compounds from agro-industrial wastes which can generate value-added products in different production chains while preserving environmental balance. The agro-industrial wastes commonly indicated in the literature correspond to the production chains of coffee, cocoa, wood, processed foods, and fruit (grapes, apples, limes, cranberries, lychee, avocado, noni, pineapple) [57,58]. This last category covers considerable amounts of residues due to the wide range of food products demanded by the market through the processing of fruit crops. It is estimated that more than 50% of the mass of the fruit is used in obtaining beverages, juices, concentrates, jams, sauces, and minimally processed products, with the remaining percentage corresponds to agro-industrial waste [59]. One of the most important crops is grapes, with an annual production of 74.3 million metric tons, where it is estimated that between 15% and 20% is generated as waste. In the case of blueberries, it has been considered that 96% of mass is used for manufacturing in the industry, in sour cherry about 85% used for the production of juices jams, fruit juices and frozen fruit [59]. From these processes peels and seeds are discarded as the main by-products, which are not edible but are rich sources of bioactive molecules [60].

Other types of waste are from fermentation processes in the production of cocoa and coffee. In 2017, the international cocoa organization reported the production of 600 tons of cocoa shell [59]. The wastes generated during forestry activities, such as skins, shells, husks, leaves, flowers and stems, contain bioactive compounds [45]. Commonly they have been used in organic fertilization, composting, energy generation, incineration, animal feed and landfilling [14]. The latest studies on obtaining bioactive compounds have been directed to the compositional and nutritional analysis of new sources of isolation from *Receptaculum nelumbinis, Acer truncatum* (leaves, stems and bark), *Euterpe oleracea* (seeds), *Zanthoxylom bungeanum* (pericarps), *Hawthorn crataegus* (Flowers, leaves), *Coniza banariensis* (leaves), *Vulgaris monocarpa* (leaves and roots), *Rhodiola rosea* (rhizome), *Prunus spinosa* (flowers), *Elsholtzia ciliata, Fagopyrum tataricum* (flowers leaves, stalk and roots), among others.

The search for natural sources of antioxidants and other biological properties for the benefit of human health has been a priority. One of the most important benefits reported in the literature is antiproliferative activity against cancer cells and the ability to scavenge free radicals [61,62,63]. However, in the literature, information on procyanidins from agro-industrial wastes, their distribution, yields in extraction, and biological activity need to be summarized and specified.

### 3.2. Ocurrence of Procyanidins in Agro-Industrial Wastes and their Biological Potential

Procyanidins and their monomers have been identified in several agro-industrial wastes from the food processing industries such as cocoa, berries, grapes, apples, litchi, blueberries, plums, avocado, nuts, tea leaves, coffee, cinnamon, peanut, leguminous plants, and several other wastes, as illustrated in Table 1 [38,44,64,65,66,67,68,69,70]. Among the main parts of the plant that are considered agro-industrial wastes and do not conflict with human food include leaves, flowers, stems, roots, bark, skin, pomace, pulp, and seed. These compounds have been found in a high percentage in seeds and berry skins. In Saskatoon berry peel, 53% of the total polyphenols are polymeric procyanidins. Moreover, the highest antioxidant activity was found in the peel, which was 16 and 5 times higher than in seeds and pulp, respectively [71]. Studies have focused on the characterization and quantification of bioactive components in baobab, a tree of African origin, concerning obtaining procyanidins; yields corresponding to 100.1 mg/100 g were reported [72]. Similar studies were carried out by Tembo et al. [73], who demonstrated that the antioxidant activity was correlated with the total phenolic content, procyanidin B2, and vitamin C.

Research indicated the presence of procyanidins in the pericarp of lychee, a residue that represents 15% of total fresh fruit weight. Miranda-Hernández et al. [74] reported percentages between 65–70% using methanol and acetone (70% *v*/*v*) as the solvent for extraction. Wong-Paz et al. [75] extracted procyanidins from coffee pulp with a concentration of 98.6% in extractions with acetone (70% *v*/*v*). Luo et al. [76] developed a method for obtaining high purity procyanidins (90%) in grape skins and seeds, achieving maximum yields of 4.1 and 13.6 mg, respectively. Other authors reported a yield of 3.94 mg of procyanidin (catechin equivalents)/gram of dried sea buckthorn seed [77]. The procyanidin content may vary according to the origin and variety of the plant material. For example, in coffee hulls, the highest procyanidin content was found in samples of the Robusta variety from India and Arabica from Mexico with 534 and 260 μg/g, respectively [78]. Authors suggest that the differences found may be due to factors such as geographic location, average temperature and irradiation level [79].

On the other hand, another topic of interest is the correlation of biological activity with the structural and chemical characteristics of procyanidins. In previous studies, the correlation of molecular weight and antioxidant activity of phenolic compounds from *Choerospondias axillaris* peel residues was determined by cellular and chemical tests, with a decrease in this biological activity occurring with an increase of molecular weight in cell assays with proanthocyanidins, while for spectrophotometric chemical assays, an increase in this variable was observed [80]. Other works report different biological activities of extracts from *Tectaria coadunata* rhizomes obtained with methanol and ethyl acetate, finding inhibitory enzyme activity (amylase and tyrosinase) associated with degenerative diseases and anticancer activity in ethyl acetate fractions. Anti-invasive and anti-proliferative activity in pancreatic cancer cell lines was also found. The highest procyanidin yields were found in the polymeric (85.46 mg/g) and trimeric (57.79 mg/g) fractions corresponding to the extractions performed with methanol and ethyl acetate, respectively [81]. A recent finding in laurel wood extracts demonstrated that B-type dimer and A type trimer procyanidins exhibited antimicrobial and antibiofilm activity [82]. Procyanidin of *Annona crassiflora* fruit peel show antiglycation capacities [83]. From *Vaccinium meridionale* Swartz pomace was obtained A-type procyanidin, demonstrating its effectiveness in the control of pathogenic bacteria [84].

Other studies suggest that the biological activities of these compounds depend on their structure, degree of polymerization, and their degree of galloylation [109]. The degree of polymerization determines their bioavailability and their ability to be adsorbed into the blood. Some studies indicate that oligomeric procyanidins are more absorbable than polymeric procyanidins [110,111,112]. Antioxidant activity has been reported to depend on the presence of the catechol group of some procyanidins, which acts as a donor of hydrogen atoms to free radicals. Other authors indicate that such activity depends directly on the number of hydroxyl groups present in the structure, which increases with the polymerization of the molecule [113,114]. In a previous study, the antidiabetic activity of oligomeric procyanidins types A and B from litchi pericarp was evaluated in tests with mice, finding the best results in type A procyanidin [115]. Oligomeric procyanidins are more effective in capturing free radicals and superoxide anions, as well as in weight control and blood glucose regulation in tests with mice [12,116]. In polymeric procyanidins, it has been verified that a protective effect against oxidative damage in hepatic cells occurred, as well as a reduction of cholesterol and fatty acid in diabetic mice [117,118]. Other studies indicated that procyanidin C1 from grape seed could prevent neurodegenerative diseases, and its antioxidant activity was verified [119].

Procyanidins are of economic interest due to their antioxidant [120], anti-inflammatory [121], antidiabetic [122], anti-aging, neuroprotective, cardioprotective [123], antiviral, and antimicrobial effects [124,125]. These biological properties allow the generation of value-added products for the pharmaceutical, food and cosmetic industries [109]. Table 2 summarizes some relevant advances in the biological activity of procyanidins from different types of agro-industrial wastes. Early studies demonstrated that grape seeds, which are source of C1-type procyanidins, showed a neuroprotective effect [119]. Forestry waste such as leaves, roots, and bark from *Annona muricate* [126], *Paullinia pinnata* [127] and *Albizia odoratissima* [128] have an inhibitory effect in lipid peroxidation, pathogen growth of plants and cancer cell proliferation, respectively. Other biological activities such as anti-VIH, anti-inflammatory, antiwrinkle effects are reported for *Cinnamomum zylanicum* [129], coffee pulp [130], and cocoa pods [46], respectively. Commercial sources obtained from grape seed have the potential for treatment of cardiovascular diseases and obesity control [131].

## 4. Extraction of Procyanidins from Agro-Industrial Wastes

Extraction methods have been used to recover bioactive phytochemicals from natural sources, which are of interest in human health. These methods were previously required to separate, purify and analyze bioactive compounds of plants [163] and are referred to in the literature as conventional or traditional or classical methods, among them being maceration (M), percolation (P) and successive solvent extraction (SSE). These methods have been widely used in the extraction of procyanidins. Some authors report procyanidin extraction from different food production chains, for example, raw cacao and blueberry [164,165]. However, researchers have proposed alternative methods that allow higher compound yields, process efficiency, and lower solvent use. Efficiency is an important factor in choosing the extraction method, being related to the recovery of compounds and biological stability of compounds, time, and energy-saving during the extraction process. Efficiency depends on the conditions of extraction, which could affect the structure of compounds as location and distribution of hydroxyl groups, terminal and extension units, interflavanic bonds, and interactions with other compounds [166,167]. This review focuses on eco-friendly recovery methods of proanthocyanidins, procyanidins, and monomeric and polymeric forms through UAE, MAE, supercritical fluid extraction (SFE), PLE and subcritical water extraction (SWE) (Table 3).

### 4.1. Ulltrasound-Assisted Extraction

Ultrasound-assisted extraction (UAE) is a simple method with short duration and has little effect on the environment. It is considered an emerging technology and better than classical methods due to rapid mass production [177]. This type of extraction has several applications in various industries (agro-industrial wastes) to obtain polyphenols such as procyanidins [107]. For the extraction of procyanidins from by-products of wine processing, UAE has been used to study its effect on the compounds’ structure and biological activity.

Extraction obtaining bioactive compounds by the ultrasound method from agro-industrial residues has been widely reported [164,179]. However, few reports of procyanidin extraction have been reported in the last five years, and most studies have focused on the evaluation of different extraction conditions on the yields of bioactive compounds [164,180,181,182,183,184,185,186]. The mechanism of extraction occurs by mechanical vibration through waves that penetrate a liquid system and form gas bubbles [187]. These bubbles are affected by the acoustic cavitation phenomenon, which leads to their collapse. Thus, there is an increase in pressure and temperature in the medium that creates an ultrasound micro-jet in the solution [188].

Grape seeds are rich sources of oligomeric procyanidins, rather than polymeric procyanidins, which represents an advantage for different industries. The ultrasound technique was used to study the depolymerization of procyanidins, finding considerable increases in the content of polymeric procyanidins, oligomeric and catechin monomers, corresponding to 41%, 35% and 49%, respectively [189].

Researchers also used this technique by varying the frequency of the test (45 and 20 kHz), obtaining higher polymeric procyanidin concentrations at 20 kHz. The breakdown of procyanidins was expected in tests with the frequency change. However, at 45 kHz the concentration of procyanidins decreased, due to obstructions in the mass transfer that occurred during the bubble formation. The improved antioxidant activity in treatments with ultrasound was associated with the hydroxyl groups, which were identified by Fourier transform infrared spectroscopy (FTIR) analysis, and could be generated by the rupture of links between procyanidins with polysaccharides or proteins [189].

A modification to the UAE is called the high-intensity ultrasound (HIUS) technique that is characterized by working at high intensity and low-frequency conditions. HIUS is attractive because of production costs, simplicity, reduced extraction times and small environment effect. Applying ultrasound of high intensity on *Araticum* peel extracts increased antioxidant activity of procyanidins A and B. These the results were achieved with short extraction times (0.5–5.0 min) [190].

Recently, combination with cavitation methods using negative pressure has been proposed to increase the yields of procyanidins, by increasing ultrasonic power between 0.2–0.35 W/cm^2^. A combination of nitrogen pressure on cavitation, collision of bubbles, cell disruption, and transfer of compounds in the extracting medium during extraction improved the yields of compounds, [164,191]. Other process conditions could affect the biological activity of procyanidins, such as vegetal material concentration, type of solvent, temperature, stirring speed, and time stirring [192,193]. Improvement of the bioavailability of procyanidins for pharmaceutical purposes has led to the development of methods for controlling the particle size of the compounds. To this goal, ultrasonic methods have been combined with the precipitation of antisolvents, with successful results under the optimal parameters of power, stirring speed, stirring of 620 W, 760 r/min, 14 min and 0.3 mg/mL, respectively, which allowed increase antioxidant activity. This may be due to the phenomenon of collision of particles by cavitation and a decrease in the agglomeration of crystals [192].

Recent advances in UAE have been explored using a macroporous resin to improve the purification and recovery of bioactive compounds, including procyanidins. The combination of these methods helps to increase extraction yield and reduce process times. The main factors that influence the quantification of the extraction process are the structure, polarity, and size of a bioactive molecule, type of resin, solution concentration, interaction resin-molecule, and ultrasound power [194]. A previous study confirmed that the employed resins (HPD-500) in ultrasonic treatments, when applied at 270–540 W for 15 and 5 min, respectively, increased adsorption capacity and mass transfer of procyanidins from baobab fruit pulp, as compared to treatments without use of ultrasound. In this study, procyanidins B2 and C1 were identified and quantified in samples treated at high power sonication (540 W) and short exposure time (5 min), with concentrations corresponding to 751.34 ± 32.76 mg/100 g dry matter (DM) and 566.38 ± 10.78 mg/100 g DM, respectively [193]. Limwachiranon et al. [180] reported procyanidin concentrations of 20 mg in lotus seed extracts using mixed solvents (acetic acid, acetone, and water).

Another type of combination with the ultrasound technique is the use of enzymes. Research has been carried out to obtain more available bioactive compounds using enzymes in ultrasound extractions using methanol as a solvent. Martins et al. [149] studied the biotransformation of condensed tannins through the enzymatic hydrolysis of tannase alone, pectinase plus cellulase, or a mixture, in white, red and mixed of grape pomaces, which were obtained from Brazilian wine production. The content of condensed tannins decreased in the enzymatic treatments with respect to the control treatment (without enzymes) and had variations in the different grape pomaces. The best results were obtained in red grape pomace in a treatment with pectinase-cellulase (21.5 mg catechin equivalents (CE)/g DM). The main polyphenolic compounds found were catechin and procyanidin B2, catechin standing outfor all treatments, with range of values for catechin and procyanidin being from 575–2009 and 166–1071 mg CE/g DM, respectively. The highest values of these compounds were observed in the grape pomace network, although they were not affected by enzymatic treatments.

In another study, isolation of procyanidins from lychee pericarp by ultra-high-pressure (UHP) extraction was compared to UAE and extraction with ethanol (ECE), to evaluate the polyphenolic profile and antioxidant activity of samples dried in an oven at 80 °C for 36 h. Polyphenolic compounds such as procyanidins A2, procyanidin B2, epicatechin, isoquercitrin and quercetin-3-rutinoside-7-rhamnoside were identified. The B2 procyanidins content was 1.13, 1.21 and 1.29 mg/g in ECE, UAE, UHP, respectively. However, the content of the A2 procyanidin was higher, with values of 4.46, 4.68, 4.97 mg/g in the same extraction treatments. These results were correlated with antioxidant activity tests, where the content of total polyphenols and antioxidant activity increased when UHP was used. Although studies have confirmed the presence of lychee procyanidins, few studies have focuses on the extraction of these compounds. The authors state that the yields of polyphenols could be improved with the adjustment of temperature and pressure in the extraction processes, since compounds such as anthocyanins are sensitive to heat, and in the case of procyanidins they can be polymerized at high temperature and pressure [195].

On the other hand, procyanidin oligomers have been extracted and purified mainly from by-products of the wine industry such as grape seeds. Due to their availability and cost, the extracts have been marketed in the food industry, especially the dietetic and supplementary market. Procyanidin polymers are macromolecules generated in these purification processes, for which extraction methods have been developed that allow their depolymerization and reduction in molecular size as catechins or oligomers. Structural characteristics and antioxidant activity of procyanidins and their derivatives in methodologies with an ultrasound bath and by a probe were evaluated. The conditions of both extraction methods were varied; the first with continuous and degas mode, and the second with 30% and 70% of amplitude in pulsed mode. The analyzes show the presence of 85% of polymeric procyanidins and 2.5% of monomeric and oligomeric flavan-3-ols. Increases in the molecular masses of these compounds were also observed when using an ultrasonic bath in continuous mode. In the MALDI-TOF analyses, type B procyanidins were observed, and the best results of antioxidant activity were obtained in the probe assays at an amplitude of 70% and a procyanidin concentration of 0.01%, while for the assays carried out in the ultrasonic bath antioxidant activity was better at higher concentration of procyanidins. The data obtained on antioxidant activity were positively affected compared to controls. The authors suggest that the ultrasound method may constitute an effective strategy to modify the structure of polyphenolic compounds in grape seeds, allowing the formation of procyanidin oligomers and polymers with antioxidant activity, that apparently could happen by the breaking of linkages with proteins and/or polysaccharides [189].

Tannin contents were reported by Kim et al. [196] in grape skin and seed ultrasound-assisted extractions using methanol, ethanol and acetone at different concentrations (10, 70 and 70%, the latter with HCl addition). The highest content of these compounds was observed in seeds extracted with 70% acetone (acidified) at 14.72 mg/g. The identified compounds were monomers and dimers of tannins such as catechin, epicatechin, epigallocatechin, procyanidin B1 and B2, procyanidin B1 being the main compound extracted for all trials. The highest content was found in skin (1076 mg/kg) and seed (1741 mg/kg) using extractions by acetone and methanol at 70%, respectively. The authors indicate that the content of the type of tannin (monomer or dimer) extracted depends on the type of solvent used. In this study, methanol was more efficient for the extraction of condensed tannins as procyanidins (dimers), while molecules of lower molecular weight, such as catechins (monomers), were extracted mainly with acetone and methanol, both at 70%.

There is extensive information on the extraction of polyphenolic compounds from winemaking pomace and marc. However, few works have focused on the use of other sources such as seedless table grape residues for the optimization of extraction parameters such contact time and solid-to-solvent ratio in UAE and MAE using mixtures of water and acidified ethanol. Crupi et al. [197] used water/ethanol/phosphoric acid (70:30:1) as solvents in UAE and MAE to recover phenolic compounds of seedless table grape residues. The main polyphenolic compounds found were procyanidin B1, procyanidin B2, (+)-catechin, peonidin-3-O-glucoside, quercetin-3-O-rutinoside, quercetin-3-O-glucoside, among others.

In processing of cranberry juice (*Vaccinium macrocarpon*), three by-products are obtained skin, seed and flesh, which are called “cranberry pomace”. The use of wastes for procyanidin extraction is an alternative to reduce the costs of blueberry juice production. Researchers have evaluated the adsorption and desorption capacity of procyanidins by using different amberlite resins (XAD-7HP, XAD-761, XAD-16N, XAD-1180, FPX-66) coupled to UAE, to study if this new method is suitable for the separation and concentration of these compounds. The results showed that the adsorption and desorption capacity of procyanidins on the resin were higher with the resin XAD-7HP. The adsorption of procyanidins on the resin was most marked between the times 600 and 800, with a maximum adsorption value of 52.2 mg/g resin. XAD-7HP resin was also used to evaluate the desorption capacity of procyanidins using different solvents (30%, 50%, 70%, 95% ethanol and 70% acetone), where the highest procyanidin desorption value was found with 95% ethanol (>250 mg/g resin), the lowest desorption capacity was found with 30% ethanol and 70% acetone [198].

Procyanidins have also been extracted from cranberry leaves by negative pressure cavitation (NPCE) and its combination with UAE. This technique was called U-NPCE by the authors. The effects of ethanol concentration, ultrasonic power, temperature, extraction time, negative pressure and solid/liquid ratio were evaluated. The authors reveal that there are still no reports indicating a solvent capable of simultaneously extracting all phenolic compounds; however, methanol followed by ethanol is more efficient for this purpose [199]. In this study, ethanol was chosen because it is food grade and was evaluated at concentrations between 40 and 90%, and for ultrasonic power and negative pressure it was evaluated at 0.3–0.4 W/cm^2^ and −0.06 to −0.08 Pa, respectively. Extraction yields of procyanidins, flavonoids and the total content of polyphenols were influenced by ethanol concentration. At 40% and 70% of solvent, the total content of polyphenols and procyanidins had higher values of 300 mg gallic acid equivalents (GAE)/g DM and 200 mg CE/g DM, respectively, which were obtained at a 70% concentration, while for concentrations between 80 and 90%, extraction yields were decreased. For the ultrasonic power parameter, an increase in extraction yields between 0.2 and 0.35 W was observed, and at the latter value the maximum yield of total polyphenols, procyanidins and flavonoids, was obtained. The temperature and extraction time positively affected the yields of the compounds studied in a range of values from 5 to 15 min, and from 30 to 50 °C. At these ranges, a gradual decrease was observed for the extraction time, while the temperature remained stable. Negative pressure significantly increased the yield of total polyphenols and procyanidins between −0.04 and −0.07 Pa. The same behavior was observed for a solid/liquid ratio between 1:10 and 1:30 g/mL. The variables ethanol concentration, ultrasonic power, and negative pressure were optimized by response surface methodology and evaluation of the bioactivity of the substances evaluated. From these results it was determined that the U-NPCE method was the most suitable for extraction of phenolic compounds such as procyanidins and flavonoids, especially those sensitive to heat, due to its high yield, bioactivity and shorter treatment time [164].

### 4.2. Microwave-Assisted Extraction

Microwave extraction (MAE) is a technique used for extraction of polyphenols such as condensed tannins and flavonoids. However, studies of procyanidins, catechins, and their structural differences still need to be updated [185,200,201,202,203,204,205,206]. MAE use solvents with a high dissipation factor (tan δ) or high polarity, such as methanol or water [207]. The mechanism consists of the transfer of heat to solvent by frequencies in the range of 300 MHz to 300 GHz, facilitating the disruption of cell wall and cellular structures. The interaction between solvent molecules and compounds released increases by the formation of pores that allow rapid mass transfer resulting in an efficient extraction process [208]. The results depend on factors such as concentration, volume, and chemical characteristics of the solvent and the cell wall [209]. The advantages of this technique are high-quality substance recovery, low use solvent, fewer plant materials, moderate time-extraction and paid energy transfer [173,184,210]. There are reports of other factors, such as extraction temperature, pressure, pH, solvent concentration and particle size, which influence choice of extraction method and solvent. However, the target compounds determine this selection, since the extraction process is specific for each plant material [91,209,211]. The variability of the food matrix and the process variables promote the selection of optimal conditions [212]. Various factors influence on the yield of the substances using this technique include temperature, time, type of solvent, ratio (solvent/solid) and power. Authors suggest response surface methodology (RSM) could reduce experiment size [157,213].

To increase extraction yields, process conditions could be modified. An example is the technique of microwave superheated water extraction (MWE) [210]. Previous findings revealed interactions between nonextractable procyanidin (native and oxidized) and polysaccharides in apple pomace using acetone (60%) and water as solvents with extraction temperatures above 100 °C for 2 and 5 min in each treatment [173]. Previous work was reported in extractions of microwave-assisted seed grape proanthocyanidin yield with recovery rates of 30.7 mg g^−1^ and 99.3%, respectively, compared with traditional extractions. This technique was carried out in two aqueous phases: acetone and ammonium citrate [211]. Proanthocyanidins extraction from apple pulp by MAE was evaluated and could improve yield and reduce extraction times.

The surface response methodology obtains the best extraction yields with the least use of solvent, energy, and time [214,215]. Authors have determined the optimal conditions in extraction processes from residues (bark) of the tree species from *Acacia mollissima*, where condensed tannin concentrations correspondimg to 74 mg cyanidin/g bark were achieved using 20% of methanol in water, 182 W and 3.66 min of time exposure [201].

### 4.3. Supercritical Fluid Extraction

The method of supercritical fluid extraction (SFE), is called supercritical CO_2_ extraction (SC-CO_2_) when CO_2_ is used as a unique solvent [216]. It has potential food applications, roles in pharmaceuticals manufacturing and polymers, and is a potential tool in separation and purification of chemical compounds and natural substances with antioxidant potential [211,214,217]. Some studies have reported results about flavonoids [218,219,220], but few are specific for procyanidins from agro-industrial and agroforestry residues [221,222,223]. This technique cannot be used with a solvent such as n-hexane, chloroform, and dichloromethane. Carbon dioxide can be used with compounds which are easily degradable by temperature, since their critical points of temperature (31 °C), and pressure (74 bar) is low; besides it is inexpensive, possess low viscosity, polarity, and reactivity, is nontoxic, nonexplosive and safe for use in food [224]. CO_2_ is useful in nonpolar and slightly polar substance extraction and, in the case of polyphenols other alternatives have been proposed to improve solvation properties and yields, such as CO_2_ in mixtures with ethanol (EtOH) and water [225,226]. The process should be designed to take into account that water in supercritical conditions is dangerous [221,225].

Temperatures and pressure monitoring makes the extraction process highly selective, and adjusting these variables obtains bioactive compounds without thermal degradation. Other solvent properties, such as volatility and surface tension are key to produce specificity for each process [227]. Some variables should be taken into account to control processes, including solvent flow rate, temperature, pressure, time, and the features of material [179,228,229]. Another important factor is equipment cost, which affects manufacturing cost. It is crucial to evaluate cost-benefits and extraction yields for future applications [230].

Authors have suggested that SFE is better than traditional methods in compound extractions with biological activity from wastes [231]. In addition, high recovery of compounds from mixtures with solvent extract can involve improved solid/liquid contact through of swelling of the solid sample or semi-solid (matrix), and can involve a low proportion of solvent [107,222]. Other researchers have found that proanthocyanidins were obtained of 139.7, 123.8, 309.3 mg catechin/100 g dried matter for monomeric, oligomeric and polymeric fractions, respectively, with mixtures of pure carbon dioxide, carbon dioxide-water (15%), and water-ethanol (15%) using grape marc at high concentrations, The authors attributed this to antioxidant activity obtained for this treatment (2649.6 mg α-tocopherol/100 g dried matter), which were greater compared to methanol extraction [227]. In a similar study, apple peel was extracted at 50 °C and 25 MPa with a mixture of CO_2_-EtOH (25%). This was correlated strongly with antioxidant activity in the presence of catechin, epicatechin, and procyanidin B [231]. Methanol (40%) was used for the modified SFE process. Three steps for the recovery procyanidin monomers were proposed, consisting of a cycle with pure CO_2_, followed by a cycle with methanol (40%)-CO_2_ and finally with pure methanol. The best recovery rates of catechin (77%) and epicatechin (79%) were achieved in the second step with 60 min of exposure [223]. Other forms of grape pomace extraction, including the coupling of ultrasound techniques with supercritical fluids, has been proposed. The maximum concentrations proanthocyanidins were achieved by SFE, which correspondedto 282.8, 167.4, and 360.3 (mg catechin/100 g dried matter) for monomeric, oligomeric, and polymeric fractions, respectively. Regarding monomeric fractions, the extraction by ultrasound was 10-fold lower than by SFE [232].

### 4.4. Pressurized Liquid Extraction

Pressurized liquid extraction (PLE), also called as accelerated solvent extraction (ASE), is considered a clean and green technology that generates by-products with added value from different natural sources [124,216,233,234,235]. However, few studies have been found concerning procyanidin extraction [236], though some works report total flavonoid content [228]. PLE has advantages over conventional extraction, such as the use of short exposure time and low solvent consumption. A range of pressure is employed (4 to 20 MPa) to keep solvent in a liquid state at high temperature when operating conditions are above boiling [219].

This process takes place in a closed and inert system at high temperatures, allowing rapid mass transfer and increasing dissolution of the plant material in the solvent [229]. The molecular interactions into the sample matrix are affected by high temperatures, surface tension and viscosity of the solvent. Polar substances and thermally sensitive subtannces have been extracted successfully with water and ethanol. The most common solvent used in PLE is water, being non-toxic, non-inflammable, and having a low cost [237]. According to operating conditions, the procyanidin content may vary. Studies have reported interactions between temperature, pressure, and/or time extraction. Okiyama et al. [29] performed extraction kinetics with cocoa bean shell at 60, 75 and 90 °C for 50 min and using 10.35 MPa. The highest yields of procyanidin were obtained at 90 °C, but this content decreased after 30 min. Researchers deduced possible changes in the matrix-solute. Mustafa and Turner [237] and Wijngaard et al. [238] indicated that the use of PLE did not increase the extraction of bioactive compounds with respect to solid-liquid extraction.

The use of PLE for bioactive substances extraction from Blackberry residues was evaluated using acidified water at 100 °C, which affected negatively the anthocyanin content but promoted the increase of activity antioxidant and total phenolics yield. Authors attributed these results to the possible presence of procyanidins and other compounds; however, the latter compounds were not measured. High temperatures allow the breakdown of interactions (hydrogen bonds, Van der Waals and others), which occur between the solvent and the plant material [234]. This activity also may be a consequence of the generation of Maillard reaction products that could affect procyanidins content. Others changes in nutritional and physicochemical characteristics have been observed [228].

An interesting option is the use of enzymes with PLE to investigate compounds from crude Guarana seeds; a plant with health benefits. This work was achieved to improve concentrations of catechin (50.59 g/100 g extract) and epicatechin (31.32 g/100 g extract) pressurizing the system at 10 MPa. The results were best with treatments using water-ethanol (50% *w*/*w*) [239], and 20 times higher than a study carried out with SFE using the same plant source [240], possibly due to low affinity of CO_2_ by polar molecules such as catechins [239]. Understanding of phenomena occurring in PLE has been mathematically modeled to optimize the process, determine interactions between variables, and allow scale-up [241].

#### Subcritical Water Extraction

A modification of PLE using only water in extraction system has been reported in the literature. Different names could be found, for example, subcritical water extraction (SWE), superheated water extraction, pressurized low polarity water extraction and pressurized hot water extraction [242], but the mechanism is the same.

This method represents an alternative use of organic solvents and could reduce negative effects to the environment and risks for human health. The use of new solvents is necessary to overcome these limitations [221]. Moreover, unlike traditional methods, it does not require removal proceedings after the process, and manipulation is automated, allowing savings of time and money. It also has good selectivity with rapid extraction. Reports have shown that treatments with SWE have a manufacturing cost higher than assays with supercritical fluids (carbon dioxide) and may be better for total flavonoid content than traditional methods, and nontraditional methods such as UAE and MAE [225,243,244,245]. SWE is used in different fields to extract bioactive compounds of different polarities, obtained using water as the solvent at high temperatures and pressures [225].

The main property of subcritical water is its dielectric constant (ε), which depends on the extraction temperature. By controlling this variable, water polarity can be changed, and thus its solvation capacity. The characteristics of water according to its dielectric constant, facilitates obtaining a wide variety of byproducts.

Other variables such as exposition-time during treatment and chemical composition of plant material represent key points in obtaining target compounds with specific characteristics. The adjustment of temperature and pressure between 100 and 374.1 °C and 1 and 221 bar, can achieve changes in viscosity, surface tension, polarity and diffusivity of water, besides improving sample solubility and mass transfer [235]. In high-temperature extractions of flavonoids, it was reported that the viscosity, density and surface tension of water can influence the structural characteristics of these compounds. Ko et al. [246] revealed the efficiency of the SWE method in extractions with residues of onion skins and sea-buckthorn leaves for nonpolar flavonoids, where temperature determined the presence of hydrogen bonding in the molecule. In this study flavonoid extractions with hydroxyl groups at low temperatures were achieved.

In previous work with winemaking residues, proanthocyanidins were extracted from grape seeds at different temperatures and cycle extractions using subcritical water. Each treatment resulted in changes in structure, linkages of catechin, and antioxidant activity of the compound. The authors indicated that this strategy allowed selectivity of processes; the type of procyanidin, number of catechin units, and ubication galloylated moieties are influenced by extraction temperature and this variable can be applied individually or sequentially. High temperatures favor polymerization of procyanidins, increased procyanidin trimers and tetramers content occurring at 150 °C, whereas subsequent treatments at 100–150 °C favored procyanidins with galloylated moieties [244].

## 5. Conclusions

Agro-industrial wastes can be used as renewable sources for the extraction of procyanidins. Undoubtedly, proper selection of the solvent and extraction technique can significantly influence the yields of these compounds. In the case of solvents, those with the highest affinity for procyanidins are methanol, ethanol and acetone, in mixtures with water. The ultrasound technique is the most preferred by researchers, followed by MAE and ASE. In order to improve extraction efficiency and procyanidin yields, UAE has been combined with other methods such as MAE, UNPLE, UHP, and hot water, among others. This is because ultrasonic extraction does not use high temperatures and is environmentally friendly. In addition it could allow greater stability of the biological activity of extracted compounds.

On the other hand, procyanidins are molecules of industrial interest for their biological properties and their effect on human health. It is common to find procyanidin extracts with high antioxidant activity, which may vary according to the plant material and the geographical area of the crop origin. Different types of procyanidins have been identified in agro-industrial wastes, among them A2, B1, B2, B3 and C1. B1 and B2 being the most frequently found.

We are aware that it is necessary to promote the saving of natural resources and promote their efficient use. Therefore, here we have shown the potential of different agro-industrial wastes as cheap sources of procyanidins, among which grape seed, litchi pericarp and plant bark are the most studied sources. In this review it was found that about 90% of studies are focused on the polyphenolic profile and biological activity of different types of wastes, while a small fraction of these studies focussed only on procyanidins. Therefore, this review encourages the researcher to study cheap and easily accessible raw plant materials as sources of procyanidins.

## Figures and Tables

**Table 1:** Sources of procyanidins from agro-industrial wastes.

Plant	Waste Type	Compound Type	Concentration	Reference
*Artocarpus heterophyllus* Lam	Peel	PCB	-	[85]
*Choerospondias axillaris*		PCB2	390 mg/g extract	[86]
*Musa* AAA		C, EC, PCs: B1, B2 and B4	-	[87]
*Passiflora ligularis and P. edulis, P. mollissima*		PCs: dimers and trimers	-	[88]
*Litchi chinensis*	Pericarp	PCs: A and B	-	[74]
*Litchi chinensis* Sonn		PCO	-	[76]
*Vitis vinifera* L.	Pomace	PCs: B1, B2	4.8–4.3 ug/kg extract	[89]
*Apocynum venetum*	Leaf	PCB2	13.4 ug/mL	[90]
*Persea americana*		PCB	-	[91]
*Combretum mucronatum*		EC, PCs: B2, B5, C1, D1	-	[92]
*Moringa oleifera*		PCB7 (dimer)	-	[93]
*Litchi chinensis*		EC, PCA2	14.8–44.5 and 44.8–69.6 mg/g extract	[94]
*Psidium guajava* L.		PCs	-	[95]
*Psidium guajava* L.		PCs	-	[95]
*Vaccinium myrtillus* L.	Leaf and stem	EC and PCB2, respectively	-	[96]
*Vaccinium vitis-idaea* L.		C, PCs: A, B, dimers, trimers	-	[85]
Crataegus spp	Leaf and flower	PCO	-	[97]
*Juglans regia*	Flower	PCA		[98]
*Crataegus monogyna*		PCO	-	[97]
*Tilia* sp		PCs	-	[99]
*Trifolium pratense*		PCs	-	[99]
*Vitis vinifera* L.	Seed	PCB2	0.41–1.6 mg/g extract	[100]
*Vitis vinifera L.*		PCs: B1, B3, B6, B4, B2, B7, B5	-	[101]
*Vitis vinifera* L		PCs	-	[99]
*Euterpe oleracea*		C, EC, PCB1	-	[95]
*Amelanchier alnifolia* Nutt	Seed and peel	PCP	1189.76–2631.73 mg/100 g extract	[71]
*Cinnamomum cassia*	Bark	PCs: A, B	-	[18]
*Aronia melanocarpa*		PCs: B2, B5, C1	-	[102]
*Fagus sylvatica* L.		C, EC, PCs: B, C.	-	[103]
*Calliandra haematocephala*		CT	841 mg/g extract	[104]
*Laurus nobilis* L.	Wood	PCB2	-	[82]
*Saraca asoca*	Stem and root	PCB2	-	[105]
*Coffea Arabica*	Pulp	PCs: B2, A (trimer), C1, tetramer, pentamer,	-	[75]
*Theobroma cacao* L.	Hulls	PCs: B1, B2	0.55–0.83 and 0.23–0.9 mg/g extract	[106]
*Elsholtzia ciliata*	Aerial parts	PCB	-	[107]
*Pyrus communis*	Ripe and over-ripe pears	PCs (low DP)	-	[108]

**Table 2:** Biological activity of procyanidins obtained from agro-industrial wastes, and possible applications.

Agro-Industrial Waste	Procyanidin Type	Application Potential/Attributes	Assay Type	Biological Activity	Reference
*Alectryon oleifolius*	PCA2	Control programs of *Cyathostomin*	In Vitro	Anthelmintic activity in larval	[132]
*Cinnamomum zeylanicum* bark	PCA (commercial compound of Indus Biotech Private Limited, Pune, India)	-	In Vivo (animals)	Anti-allergic	[133]
*Rhododendron formosanum* leaves	PCC4 and cinnamtannin D1	Development of pharmaceutical products	In Vitro	Antibacterial, and pleiotropic effects	[134]
*Theobroma cacao* L. pods	PCs: dimer B1 and trimer C2	Functional cosmetic	In Vitro	Antioxidant and anti-wrinkles	[135]
*Punica granatum* L. peel	C, EC, dimer A, dimer B1, dimer B2, dimer B3, trimer A	-	In Vitro	Antioxidant, inhibition of α-glucosidase activity, lipase activity, LDL-cholesterol oxidation	[46]
*Cinamomum Zylanicum* bark	PC A (trimerics and pentamerics)	-	In Vitro	Anti-VIH-1	[8]
*Vitis vinifera* seed	PCB1	Cancer chemoprevention, antineoplastic agent, cardiovascular benefit	In Vitro/In Vivo (animals)	Inhibition of cyclooxygenase-2 and enhance prostacyclin	[9]
*Potentilla erecta* L. rhizome	PCB	-	In Vivo (animals)	Antithrombotic	[115]
*Garcinia mangostana* pericarp	Monomers, dimers, and PCO	-Ingredient in chocolate processing -Nutritional and sensory quality	In Vitro	-	[136]
*Paullinia pinnata* L. Roots	C, EC, and PCs: trimeric A, tetrameric A, and PCP.	-	In Vitro	Anthelmintic	[127]
*Theobroma cacao* L. powder	C, and PCB2	Treatment against cancer	In Vitro	Cytotoxic effect in ovarian cancer cell, impact the regular cell cycle progression of cancer and overcoming drug resistance	[135]
*Litchi chinensis* pericarp	PCO: A-type	-	In Vitro	Hypoglycemic	[137]
*Acacia mearnsii* leaves	PC: dimer B	-	In Vitro	Anti-inflammatory and antioxidant	[138]
*Larix decidua* bark	C, EC, and PCs: B2, trimer B, and tetramer B	-	In Vitro	Antioxidant	[139]
*Clausena lansium* pericarp	C, EC PC with DP up to the 20-mers.	Chelating ability	In Vitro	Inhibition on the melanogenic activity in B16 anti-tyrosinase	[140]
*Litchi chinensis* pericarp	PCA2	Therapy of liver damage	In Vitro/In Vivo (animals)	Antioxidant	[141]
*Annona crassiflora* peel	C, EC and PC B2	Treatment of diabetes	In vitro	Antioxidant, hepatoprotective effect, influence glutathione reductase activity and glutathione level.	[142]
*Vitis vinifera* seed	C, EC, and PCs: dimer B1, dimer B2, dimer B5, dimer B2 gallate, trimer C1, trimer T2, tetramer A2, unknown dimers, pentamers, and hexamers	-	In Vitro	Antimicrobial	[143]
*Triplaris gardneriana* seeds	C, PCs: dimer B, digalloylated PC PCB, monogalloylated procyanidin dimer B	Rapid biotransformation	In Vitro	Antioxidant	[144]
*Schinus terebinthifolia* Stems	PCB	Treatment of Herpes simplex virus type infections	In Vitro/In Vivo (animals)	Antiviral	[145]
*Larix gmelini* bark	PCs	Healthcare, and cosmetic products	In Vitro	Antimicrobial, affects membrane protein synthesis of *Staphylococcus aureus*	[146]
*Paullinia cupana*	PCs	Prevention and control of *Helicobacter pylori*	In Vitro	Antioxidant and gastroprotective activity	[144]
*Tamarindus indica* seed coat	Mixture of procyanidins	Treatment therapeutics	In Vitro	Antioxidant	[145]
*Vitis vinifera* pomace	PCs: dimer, trimer C1 and trimer (possible C2)	-	In Vitro/In Vivo (animals)	Antioxidant, enhancement of catalase and glutathione peroxidase activity in colon. Enhancement of superoxide dismutase activity in duodenum	[147]
Juice from Unripe *Vitis vinifera*	C, EC and PCs: B1 and B2.	-	In Vitro	Antioxidant and Anti-browning	[148]
*Annona muricata* leaves	EC, and PCs: B2 and C1	Treatments in diabetes mellitus	In Vitro	Antioxidant, inhibitory activities against advanced glycation end-product formation, pancreatic lipase, α-amylase, α-glucosidase, and lipid peroxidation	[129]
*Vitis vinifera* seed	PCB2 3,3”-di-O-gallate	Control malignant cells in prostate	In Vitro	Inhibition MAP kinase phosphatase	[149]
Commercial (*Vitis vinifera* seed)	PCs dimers	Potential in cardiovascular and obesity treatment	In Vivo (animals)	Reduces adiposity and oxidative stress in the heart	[150]
Coffea arabica L. pulp	PCs: dimers and trimers		In Vitro	Anti-inflammatory and inhibition interleukin-8 release in human gastric epithelial cells	[151]
*Trichilia catigua* bark	PCB2–8-C-rhamnoside, PCB2 (epi)-catechin—(epi)-catechin	Potential as antifatigue drug	In Vivo (animals)	Antioxidant, anticholinesterase,	[149]
*Calluna vulgaris* L. flowers	Proanthocyanidins	Food ingredient	In Vitro	Antioxidant and antimicrobial	[152]
*Persea americana* peel and seed	C, EC, and PCs: B1 and B2,	-	In Vitro	Antioxidant and anti-inflammatory	[153]
*Persimmon vinegar* pulp	PCA2	-	In Vitro	Hepato-protective effects	[154]
Stem bark of *Detarium microcarpum, Cassia siamea,* and *Guiera senegalensis*	PCB3	Anti-breast cancer agents	In Vitro	Antioxidant, and antiproliferative effects on cancer cells	[155]
Leaves and stem bark of *Ficus curtipes*	C, EC and PCs: B2 and C	-	In Vitro	Anti-inflammatory and modulation of nitric oxide synthase enzyme expression	[156]
*Fraxinus angustifolia*	C and PCB1	Regulation of signaling pathways homeostasis	In Vitro	Antioxidant and anti-inflammatory	[157]
*Nelumbo nucifera* seed epicarp	PC with of epicatechin units linked by B-type interflavan bonds	-	In Vitro	Antioxidant and anti-α-amylase	[158]
Commercial standard of Sigma-Aldrich, Merck KGaA	PCB2	-		Inhibition of angiogenesis, fibrogenesis processes, Inhibition of proliferation and induction apoptosis of human hepatic stellate cell	[156]
*Vitis vinifera* marc	C, EC, and PC trimer	Thermal stability	In Vitro	Antioxidant	[159]
Commercial of Extrasynthese (Genay, France)	PC s: A2 and B2, and cinnamtannin B-1	Prevention of urinary tract infections	In Vitro	Antiadhesive of uropathogens	[160]
*Vitis vinifera* seed and pine bark	C, EC, PCs: dimers 1, 2, B1, B2, and B5, trimers 1, 2, 3 and C1, tetramer 1, pentamer 1 and 2, hexamer 1and 2, heptamer 1 and 2, octamer, decamer, dimer gallate and dimer B2 gallate	-	In Vivo (animals)	Enhances cytokine production	[161]
*Melastoma malabathricum* leaves	PCA	-	In Vivo (animals)	Cytotoxic effect against colon cancer cells	[162]
*Albezia odoratissima* bark	PCC1	-	In Vitro	Anticancer in breast	[131]
*Feijoa sellowiana* leaves	C, PCs: dimer B1, B2 and B3, galloyled procyanidin dimer, digalloylled PC dimer	-	In Vitro	Inhibition of acetylcholinesterase and antilipase activity	[163]
Residual cake of *Pistacia vera* L.	C, EC, and PCs: B1, B2, and galloylated dimer	-	In Vitro	Antioxidant	[128]
*Vitis vinifera* seed	PCC1	Application for neurological disorders	In vitro	Antioxidant, neuroprotective	[164]
Skin, seed, skin, and bunch stem of *Vitis vinifera*	PC tetramer (crown PC), PCO, PCP	-	In Vitro	Inhibition of amyloid-β peptide	[162]

**Table 3:** Research on extraction of procyanidins from agro-industrial wastes, and future applications.

Plant/Waste	Technique	Conditions *	Type de Procyanidin or Yield	Type of Application	Reference
*Vitis vinífera*/skin	SSE and UAE	5 g/100 mL Methanol 60% Solution acidified water pH 1.5	-	Conservation of food	[168]
*Vaccinium macrocarpon*/pomace	SSE	-Hexane 40 mL, centrifugation 10 min at 10,864× *g*. -Ethyl acetate (40 mL), and centrifugation for 10 min at 10,864× *g*. -Neutralization and mixing with 20 mL of acetone/water/acetic acid, (70:29.5:0.5 *v*/*v*/*v*), homogenization for 1 min. -All treatments at 25, 40 and 60 °C.	519.3 mg of PC/100 g DM at 60 °C.	Nutraceutical, estimation method of PC	[111]
*Malus domestica*/pomace	Enzymatic maceration	Pectinex (20 mL/100 kg sample), stirring 1 h at 20 °C.	-PCs: B1 (18.7 mg/mL), B2 (80.2 mg/mL), and C1 (18.7%). -PCP: 57 and 24% corresponding to total polyphenols in pomace and fresh juices, respectively.	Production of beverages and drinks	[169]
*Betula pendula*/bark	SSE	Methanol (8%)-Water.	PC (dimer)	Isolated procyanidin glycosides (rarely found in nature)	[170]
*Vitis vinífera* (Wine waste)/seed, skin, pomace	SSE	Acetone (50%)-water, ethanol (50%)-water, Methanol (50%)-water.	PCs: A and B	Antioxidant activity	[171]
*Pinus pinaster*/bark	High pressure solvent (HPE)	-CO_2_, CO_2_-ethanol (90:10), 3 times, 323 and 303 °C, 20.3 and 25.1 MPa, 370 and 360 min; 7.6, 13.2, and 19.1 kg/s × 10^5^, solvent-to-solid mass ratio 28:1, 2:1, 20:1. -CO_2_-ethanol (90:10) 3 times, 303 °C, 25.1 MPa, 360 min; 7.6, 13.2, and 19.1 kg/s × 10^5^, solvent-to-solid mass ratio 28:1, 2:1, 20:1. -CO_2_-ethanol (70:30), CO_2_-ethanol (50:50), CO_2_-ethanol (30:70) and CO_2_-ethanol (10:90); 303 °C, 25.1 MPa, 210 min; 7.6 kg/s × 10^5^, solvent-to-solid mass ratio 28:1, 2:1, 20:1.	19.8 % (mg CME **/mg extract × 100 dried base). The best result was achieved with CO_2_-ethanol (70:30) with Flow rate of 7.6 kg/s treatments.	Improved extraction methodologies	[172]
*Malus domestica*/pomace	* 1. M, 2. PLE, 3. UAE, 4. MAE	1. Solid-solvent ratio (ethanol) 1g/1ml, stirred 1 h, room temperature. 2. Ethanol, 3 min 40 °C, 100 bar, 3 cycles. 3. Relation solute—solvent (ethanol) 3 g/60 mL, 30 min, room temperature. 4. Solid-solvent ratio (ethanol ethyl acetate or water/methanol) 1 g/20 mL, 3 s, 3 cycles, 1000 W.	PC (dimer), best results: MAE (ethanol or ethyl acetate).	Antioxidant activity	[173]
*Larix gmelinii*/bark, xilem	UAE	Bath power 250 W, Solute-solvent ratio 5 g/100 mL ethanol (50%), 0.5 h.	601.94 mg PC/g bark (North orientation)	Use of wood in industrial process.	[151]
*Coffea arabica* L./pulp	* 1. UAE, 2. M	1. Ethanol-water (70:30), water-ethanol (30:70), 100% milliQwarer, Power 100 W, 30 min, room temperature in the dark. 2. Ethanol-water (70:30), water-ethanol (30:70), 100% milliQwarer, 16 h, room temperature in the dark.	PC A (dimers and trimers) obtained with UAE.	Food supplements	[174]
*Theobroma cacao*/bean shell	PLE	Solvent-to-solid ratio 1:3, 60, 70 and 90 °C, 5–50 min, 10.35 Mpa.	Yield: 0.73 mg PC B2/g dried cocoa Shell (60 °C, 50 min)	Antioxidant activity	[73]
*Acer truncatum*/seed coat	SSE	Solution with 20 g of sample, water, ethanol (100%), aqueous acetone (70%), acetone (100%), aqueous ethanol (70%), and aqueous ethanol (40%), 30 min, centrifugation 6000 rpm by 10 min.	PC (dimer, trimer, tetramer, pentamer)	New phytochemical	[75]
*Vitis vinífera* (Wine waste)/seed, stem skin, pomace	MAE	Power 98 W, 24 °C, Ethanol (10 mL), extraction time 5-15 min, vegetal sample 1–2 g.	PC (trimer)	Formulations of food, chemical, pharmaceutical products	[157,175]
*Theobroma cacao*/bean shell	* 1. UAE, 2. Hydrodynamic cavitation (HC)	1. -Hexane, 40 °C, 15 min -Solvent-to-solid ratio 70:30 (ethanol/Water). -Solvent-to-solid ratio 30:49:21 (Hexane/ethanol/water). 2. Ethanol/Water 3000 rpm, 11 min, cycles 47.1, cycle times 5 s, residence time 5 s, adsorbed energy 6.82 KW.	PC for HC	Process design	[175]
*Malus domestica*/parenquima, skim	SSE	Hexane, methanol/acetic acid (99:1 *v*/*v*), Acetone/water/acetic acid (60:39:1 *v*/*v*/*v*).	PC with a DP 9	Formulations of juices	[176]
*Vitis vinífera*/seed	SSE	Ethanol/water (1:1, *v*/*v*), stirring 30 min in the dark.	PCB (dimer and tetramer)	Nutraceutical products	[27]
*Theobroma cacao* L./bean shell	* 1. MAE, 2. SSE	1. Power 500 W, heating rate 20 °C/min, 400 rpm, initial solid-solvent ratio 6 g/250 mL water, solid/liquid ratio 0.030, 0.045, 0.060 g/mL, extraction time 5, 15, 25 min and at 70, 85, 100 °C. 2. Solid/liquid ratio 0.045 g/mL, 100 °C, 90 min, centrifugation 5300 rpm, 25 min.	Polyphenols 35.9 mg GAE/g.	Food additives, food packaging	[177]
*Malus domestica*/pomace	SSE	-Diethyl ether/ethyl acetate (DE/AE) (1:1, *v*/*v*), Acid hydrolysis (pH 2). -DE/AE (1:1, *v*/*v*), base hydrolysis (pH 2). -Methanol (80%)-Water, 1% acid formic, exposition 2 times.	PC B2	Functional products	[178]
*Vaccinium*/pomace	* 1. PLE—Ethanol 2. PLE—Water	1. 83 °C, 15 min, °C, 3 cycles. 2. 130 °C, 10 min, °C, 3 cycles.	198.5 and 532.2 mg of proanthocyanidins/g. Recovery of dimer PCB2 (578.5 Daltons)	Processing residue at industrial level.	[176]

* The numbers correspond to the conditions in each type of extraction described in the table. ** CME: (+)-catechin monohydrated equivalents.

## Abbreviations

UAE, ultrasound-assisted extraction; MAE, microwave-assisted extraction; ASE, accelerated solvent extraction; PLE, pressurized liquid extraction; HE, hot extraction; HR, heat reflux; M, maceration; P, percolation; SSE, successive solvent extraction; SFE, supercritical fluid extraction; SWE, subcritical water extraction; MWE, microwave superheated water extraction; ASE, accelerated solvent extraction; CME, catechin monohydrated equivalents; DE/AE Diethyl ether/ethyl acetate; HIUS, high-intensity ultrasound; FTIR, Fourier transform infrared spectroscopy; CE, catechin equivalents; DM, dry matter; NPCE, negative pressure cavitation extraction; GAE acid equivalents; DP, degree of polymerization; C, catechin; EC, epicatechin; PCs, procyanidins; PCO, oligomeric procyanidins; PCP, polymeric procyanidin; CT, condensed tannins; CME, catechin monohydrated equivalents.
